# Joint Model-Order and Robust DoA Estimation for Underwater Sensor Arrays

**DOI:** 10.3390/s23125731

**Published:** 2023-06-20

**Authors:** Umar Hamid, Shurjeel Wyne, Naveed Razzaq Butt

**Affiliations:** 1 Department of Electrical and Computer Engineering, COMSATS University Islamabad (CUI), Park Road, Islamabad 45550, Pakistan; shurjeel.wyne@comsats.edu.pk; 2 Department of Engineering Sciences, Ghulam Ishaq Khan Institute of Engineering Sciences and Technology (GIKI), Swabi 23640, Pakistan; naveed.butt@giki.edu.pk

**Keywords:** array signal processing, beamforming, compressive sensing, direction-of-arrival estimation, sparse reconstruction, underwater environment

## Abstract

The direction-of-arrival (DoA) estimation algorithms have a fundamental role in target bearing estimation by sensor array systems. Recently, compressive sensing (CS)-based sparse reconstruction techniques have been investigated for DoA estimation due to their superior performance relative to the conventional DoA estimation methods, for a limited number of measurement snapshots. In many underwater deployment scenarios, the acoustic sensor arrays must perform DoA estimation in the presence of several practical problems such as unknown source number, faulty sensors, low values of the received signal-to-noise ratio (SNR), and access to a limited number of measurement snapshots. In the literature, CS-based DoA estimation has been investigated for the individual occurrence of some of these errors but the estimation under joint occurrence of these errors has not been studied. This work investigates the CS-based robust DoA estimation to account for the joint impact of faulty sensors and low SNR conditions experienced by a uniform linear array of underwater acoustic sensors. Most importantly, the proposed CS-based DoA estimation technique does not require a priori knowledge of the source order, which is replaced in the modified stopping criterion of the reconstruction algorithm by taking into account the faulty sensors and the received SNR. Using Monte Carlo techniques, the DoA estimation performance of the proposed method is comprehensively evaluated in relation to other techniques.

## 1. Introduction

The underwater acoustic propagation channel is a more harsh propagation environment than the well-studied terrestrial radio channel [1]; the higher propagation loss [2] and more severe noise conditions coupled with surface and sea-bed interference result in low SNR [1,2]. Also, the frequency-dependent signal attenuation [3] leads to limited bandwidth at longer distances [3]. A single sensor may detect the presence of a signal source but cannot determine its angle of arrival. An array of hydrophones is required to estimate the DoA for each of the targets from their received signals, provided that a signal processing block is attached with the array [4]. Such an intelligent system can utilize beamforming to estimate target bearing, resolve multiple targets, and determine target range in certain scenarios. In the case of sonar systems whether active or passive, beamforming can act as a spatial filter to allow signals incident from the desired direction to pass through, whereas interfering signals from all other directions are attenuated [5]. Hydrophone arrays are deployed in underwater scenarios to detect weak acoustic signals from single or multiple targets to estimate their bearing. In case the sources are close to one another in distance or bearing, they need to be resolved accordingly [6]. Beamforming techniques can be broadly classified as narrowband and broadband beamforming. Beamforming for a narrowband signal (i.e., single frequency) is based on phase shifting of the signal and is widely used to generate desired steering vectors based on these phase shifts [7]. Narrowband beamforming can be extended for a wideband signal, i.e., multiple frequencies) by dividing it into discrete frequency components followed by proper weighting of each frequency component [8].

### 1.1. DoA Estimation Algorithms: Background

The DoA estimation of an impinging signal is one of the most investigated applications of array processing in the context of radar, sonar, seismology, and wireless communications. In order to electrically steer the antenna beam in the desired direction, the true angle of the desired signal is assumed to be known. However, in practice, the same needs to be estimated with some acceptable inaccuracy [9]. The three major categories of DoA estimation algorithms are (i) conventional beamforming, (ii) subspace-based methods, and (iii) maximum likelihood (ML) methods [9]. In conventional methods of DoA estimation, beams are steered in all possible directions and the DoA corresponding to the peak output power is returned as the DoA estimate. Though the implementation is reasonably simple, it requires a large number of sensors to obtain an increased resolution. In conventional algorithms, e.g., Capon’s method [10], output power of the unwanted signal is minimized and on the other hand a constant gain is maintained in the desired signal direction. Then the angular spectrum is computed for all directions and peaks in the spectrum correspond to the estimated DoAs. The accuracy of Capon’s method is highly dependent on the number of sensors [10].

Subspace-based methods such as multiple signal classification (MUSIC) and estimation of signal parameters via rotational invariance technique (ESPRIT) offer increased angular resolution as compared to the conventional algorithms for DoA estimation [7]. Both techniques perform eigenvalue decomposition on the array covariance matrix and obtain a signal subspace and a corresponding orthogonal noise subspace. The MUSIC spectrum is computed for all possible directions and the peaks in the angular spectrum represent the desired signal sources based on the principle of orthogonality [11]. The ESPRIT algorithm divides the sensor array structure into two sub-arrays, i.e., identical and equal-sized with corresponding sensors in these sub-arrays separated from each other with a fixed distance. The ESPRIT spectrum is computed in a way to provide only those peaks that correspond to the incoming signal sources and hence yield better resolution [12]. These methods can provide high-resolution DoA estimates for uncorrelated signals. However, in a multipath environment (i.e., reflection and refraction of source signals), coherent signals are observed in the received data, resulting in a rank deficient covariance matrix thereby degrading the performance of these subspace-based techniques [12].

The maximum likelihood (ML) method of DoA estimation performs better than conventional and subspace methods under unfavorable conditions including low SNR, small number of observations, and multiple correlated signals [12]. However, the ML method is significantly more computationally intensive than the other methods and, like the subspace-based methods, the ML techniques also require a priori knowledge about the number of sources, which is typically not available in practical situations [12]. On the other hand, the adaptive beamforming techniques can provide better performance subject to the availability of enough data for algorithm convergence [13]. However, adaptive beamformers are sensitive to array steering vector (ASV) errors and interference non-stationarity [13]. In many practical applications the ASV errors and interference non-stationarity can occur at the same time, and hence several adaptive beamformers have been developed, to minimize the effects of these two factors, that include diagonal loading and robust adaptive beamforming techniques and their variants [13,14].

In general, the adaptive beamforming approach requires a large number of snapshots for accurate covariance matrix estimation [13]. In many practical scenarios, data snapshots are often limited and this results in a difference between the estimated sample covariance matrix and the true statistical covariance matrix, thereby causing performance degradation [13]. Adaptive beamformers based on covariance matrix reconstruction can achieve a faster convergence provided that the number of snapshots exceeds the number of sensors [13]. However, with multiple snapshots, the adaptive beamformers become computationally intensive as they collect array data, estimate and invert the sample covariance matrix, and update the beamforming weights for every data chunk received [14]. In addition, an adaptive beamforming system for large-scale arrays such as passive sonar towed arrays comprising hundreds of sensors requires a large amount of hardware and adds to software complexity [14].

In light of the aforementioned practical errors, new approaches to DoA estimation based on the emerging field of compressive sensing (CS) have been investigated in the literature [15,16].

### 1.2. Compressive Sensing (CS) Theory and CS-Based DoA Estimation

CS theory deals with the acquisition of sparse/compressible signals that can be represented with a few significant constituent components [17,18]. A signal may be sparse in its measurement domain or it can be expressed in a sparse representation using a suitable sparsifying basis such as the Fourier basis [19]. CS theory involves three important aspects: sparse signal representation, design of the measurement matrix, and the reconstruction algorithm [19]. Different types of measurement matrices, i.e., random and deterministic matrices, can be used for sparse signal reconstruction. The most commonly used measurement matrices are Gaussian matrix, Bernoulli matrix, and partial Fourier matrix, etc. [19]. CS-based reconstruction algorithms are used to recover the sparse signal from its measurement of a lower dimension [15,16].

The CS-based DoA estimation techniques exploit the fact that the signal incident on the sensor array can be viewed as a sparse signal in the spatial domain, i.e., the number of actual incident angles is much lower than the number of potential incident angles [20]. The DoA estimation problem can be formulated by dividing the angular space into *v* DoA bins, where only u≪v bins contain an actual impinging signal [15,16]. A CS-based sparse representation model can be constructed through the introduction of an overcomplete dictionary or measurement matrix A whose rows correspond to the number of sensors and its columns correspond to potential angles of incidence [19]. Then by using CS-based sparse recovery algorithms, the unknown incident DoAs can be estimated from the measurements by finding the minimum number of DoAs with non-zero impinging signals under certain constraints [21]. These CS-based recovery algorithms can be broadly classified as optimization-based methods for the convex case, e.g., the basis pursuit denoising (BPDN) algorithm [19], and for the non-convex case, e.g., the multi-resolution focal underdetermined system solver (MFOCUSS) algorithm [22], and the greedy algorithm-based approaches, e.g., the OMP algorithm [23]. In addition, a popular data-dependent covariance matrix based iterative approach, known as the iterative adaptive approach (IAA), can also provide accurate DoA estimates in the high SNR regime (SNR ≥ 0 dB) [24,25].

In many underwater deployment scenarios, sensor array systems have to face different problems such as gain and phase uncertainties [26], non-uniform noise [27], faulty sensors in the array [28,29,30], unknown source order [31,32], resolution of closely spaced sources [33,34], off-grid DoAs [35,36], availability of limited snapshots [20,23], and low SNR [37,38].

In ship-towed underwater sensor arrays, the acoustic sensors are sealed in a tube-like structure and the repair or replacement of a single faulty sensor is not an option. Hence, sensor failures become a practical problem due to corrosion and other harsh conditions in the array’s deployment scenario [39]. The array’s faulty sensors result in increased sidelobes in the array’s beampattern, which degrades the DoA estimation performance [29,40]. In [28], an improved CS-based acoustic beamformer was presented for DoA estimation by an array with faulty sensors. The algorithm is based on orthogonal matching pursuit (OMP) and the simulation results of this method showed better performance than the conventional beamforming approach. On the other hand, the authors in [30] described methods for source localization over impaired uniform linear array based on covariance matrix reconstruction. With a mechanism developed for detecting the number and position of failed sensors, these methods exploit the root-MUSIC- and ESPRIT-based techniques, respectively, to estimate the DoAs and showed better performance than the classical DoA estimation algorithms, in the failed sensors scenario.

The subspace-based DoA estimation techniques such as MUSIC and the classical CS-based recovery algorithms such as OMP require a priori knowledge of the number of sources [12,23]. In the literature, the Akaike information criterion (AIC) and the minimum description length (MDL) methods have been proposed to estimate the source number, but their estimation performance degrades with decreasing number of snapshots and decreasing SNR [31,32].

Furthermore, for two sources closely spaced in the angular domain, the desired mutually coherent condition of CS-based methods is violated in greedy algorithms such as the OMP and as a result the beamformer shows a single peak instead of distinguishing between the coherent sources. Several works have discussed different variants of the classical OMP, e.g., regularized OMP (ROMP), to improve its DoA estimation accuracy under these conditions [33,34]. Another popular greedy method is the simultaneous OMP (SOMP) algorithm for DoA estimation using multiple snapshots [19,22]. Also, it is noteworthy that in a given scenario not all true DoAs will fall on the discrete search grid points which leads to an off-grid error between the true DoA and its closest grid point. The authors in [36] presented a new computing paradigm using a CS-based technique to deal with the off-grid DoA estimation problem.

The DoA estimation problem is extensively studied under the assumption of independent Gaussian distributed sensor noise [10,11,12]. However, non-Gaussian distributed impulsive noise may also arise in some scenarios due to factors such as the equipment’s sudden impact noise, and natural or man-made electromagnetic interference and manifests in the sensor’s received noise as sharp spikes and a heavy-tailed distribution [41,42]. The DoA estimation under impulsive noise has been studied through different approaches including sparse Bayesian learning [41], non-linear similarity measures such as the generalized maximum complex correntropy criterion [43], and modeling the impulsive noise with other distributions [44].

Detection and classification of acoustic signals is a challenging task in underwater sensor array systems. Traditional underwater passive detection methods such as energy detection show a poor performance under low SNR ocean environment. Over the past few decades, the underwater environment has become more complicated as the ambient noise has increased due to increased traffic. In addition, improved acoustic technologies have significantly reduced the radiation noise of underwater targets. Subsequently the existing underwater passive methods are facing serious challenges in detecting targets. This has resulted in investigating advanced DoA estimation algorithms offering better performance, especially under low SNR conditions [37,38].

The classical subspace DoA estimation algorithms and adaptive beamforming approaches require a large number of snapshots for covariance matrix estimation and algorithm convergence, respectively. Their performance degrades significantly when limited snapshots are available [20]. In comparison with conventional beamforming methods, the CS-based algorithms can work with a limited number of snapshots or even a single snapshot and provide higher angular resolution. Unlike adaptive beamforming methods, these algorithms do not take the inverse of the array covariance matrix. Also, with multiple snapshots, these algorithms can outperform high-resolution methods, e.g., MUSIC in the presence of coherent sources and low SNR conditions [15,16]. In view of these advantages, the researchers are actively investigating the performance of CS-based DoA estimation algorithms, in the presence of the aforementioned practical errors [23,33,34].

### 1.3. Contributions and Organization

In view of the aforementioned works and to the best of the authors’ knowledge, the problem of CS-based DoA estimation with faulty sensors and low SNR conditions has not been jointly investigated under unknown source model order, which is addressed in this work. Specifically, our main contributions are listed as follows.

- A modified OMP algorithm for DoA estimation is proposed that does not require a priori knowledge of the source order. The algorithm is shown to work for both the single- and multi-snapshot cases and the corresponding estimators are termed as Model-Order and DoA Estimator using OMP (MODE-OMP) and MODE-SOMP for the single- and multi-snapshot cases, respectively.
- The proposed DoA estimators also use a modified stopping criterion to incorporate the effects of faulty array sensors and low received SNR to furnish accurate DoA estimates under these conditions.
- The DoA estimation performance gain of the proposed algorithms is shown in relation to other notable CS-based techniques in terms of the root mean squared error (RMSE) of the DoA estimates and the probability of target resolution, for different error scenarios relevant to underwater acoustic array deployment.

The rest of this paper is organized as follows. Section 2 describes the system model including the CS-based reconstruction for DoA estimation. Section 3 elaborates practical error models encountered in underwater sensor array systems. Section 4 provides details of the CS-based proposed DoA estimation algorithm. Section 5 presents the DoA estimation performance of the proposed method in comparison with existing schemes. Finally, Section 6 concludes this work.

Notation: The boldface lowercase letters represent the vectors and the boldface uppercase letters represent the matrices. The superscripts ${\left(.\right)}^{-1}$, ${\left(.\right)}^{T}$, and ${\left(.\right)}^{H}$ denote the matrix inverse, transpose, and Hermitian transpose operators, respectively. The symbols ${\left||.|\right|}_{1}$ and ${\left||.|\right|}_{2}$ denote the $l_{1}$-norm and $l_{2}$-norm, respectively. Furthermore, the symbol |.| denotes the absolute value. $C^{M\times N}$ denotes an M×N matrix with complex-valued entries. Finally, <x,y> denotes  the inner product of vectors x and y, and E[.] stands for the statistical expectation operator.

## 2. System Model under the CS Framework

This section explains the system model considered in the analysis.

### 2.1. Signal Model

**Figure 1:**
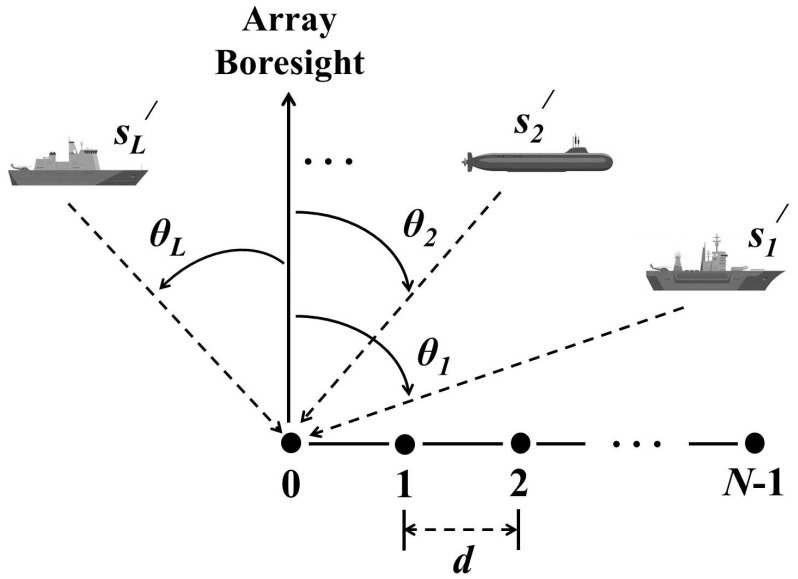
System model.

Consider a uniform linear array (ULA) with *N* sensors and L<N plane waves from narrowband sources that are incident at the ULA with respective angles $\theta_{l}$, l=1,2,…,L (the case L>N pertaining to more sources than physical sensors is not considered here but can be addressed through specially designed sparse array structures such as nested arrays [45] or co-prime arrays [46] to achieve the required increase in degrees of freedom). For the considered ULA, the $\theta_{l}$ can be unambiguously estimated in the range $-\pi /2\leq \theta_{l}\leq \pi /2$. As shown in Figure 1, adjacent sensors are separated by a fixed spacing d=λ/2 where λ is the signal wavelength. At the time instant *t*, $t=1,2,\ldots ,T_{s}$, where $T_{s}$ is the total number of snapshots, the received signal vector $x\left(t\right)\in C^{N}$ can be expressed as

(1) $$x\left(t\right)=\sum_{l=1}^{L}a\left(\theta_{l}\right)\;s_{l}^{\prime }\left(t\right)+w\left(t\right)=A^{\prime }\;s^{\prime }\left(t\right)+w\left(t\right),$$

where $s^{\prime }\left(t\right)={\left[s_{1}^{\prime }\left(t\right),s_{2}^{\prime }\left(t\right),\ldots ,s_{L}^{\prime }\left(t\right)\right]}^{T}\in C^{L}$ represents complex amplitudes of the incoming signals, and $w\left(t\right)={\left[w_{1}\left(t\right),w_{2}\left(t\right),\ldots ,w_{N}\left(t\right)\right]}^{T}\in C^{N}$ is the independent and identically distributed complex Gaussian noise at the sensors with zero mean and variance $\sigma^{2}$. Also, $a(\theta_{l})$ is the array response vector for the signal with incoming direction $\theta_{l}$ and is expressed as

(2) $$a\left(\theta_{l}\right)={\left[1,e^{\left(\frac{-j2\pi d\sin(\theta_{l})}{\lambda }\right)},\ldots ,e^{\left(\frac{-j2\pi \left(N-1\right)d\sin\left(\theta_{l}\right)}{\lambda }\right)}\right]}^{T}.$$

Furthermore, $A^{\prime }=\left[a\left(\theta_{1}\right),a\left(\theta_{2}\right),\ldots ,a\left(\theta_{L}\right)\right]\in C^{N\times L}$ is the array response matrix.

A malfunctioning *i*th sensor in the ULA is assumed to provide no useful information such that its corresponding *i*th row in the array response matrix $A^{\prime }$ can be set to all zeros [28,30]. Furthermore, in many practical situations, the source order *L* is unknown a priori and also needs to be estimated using some estimation techniques such as the AIC [31] and MDL [32].

### 2.2. CS-Based DoA Estimation for a Single Snapshot

For the single-snapshot case, the dependence on *t* can be dropped to simplify notation and (1) can be expressed in matrix form as

(3) $$x=A^{\prime }\;s^{\prime }+w.$$

The DoA estimation problem is to find the DoAs, $\theta_{l}$, by utilizing the received signal, x. As stated before, performance of high-resolution DoA estimation algorithms depends on some statistical properties, e.g., the covariance matrix, which in turn requires a sufficiently large number of snapshots. However, in some cases, it is difficult to obtain sufficient snapshots, e.g., a high-dynamic target. Hence, the performance of all high-resolution DoA estimation algorithms degrades with a small number of snapshots. Fortunately, CS-based algorithms can be used to estimate the DoAs, with limited or even a single snapshot [20,23].

In general, CS theory considers the problem of estimating the sparse signal vector z from linear measurements of the form y=Hz+e, where H is a measurement matrix and e is the noise vector. The number of rows of H is far smaller than its number of columns. If z is sparse or compressible and H satisfies the incoherence condition [17,18], then CS-based algorithms provide an efficient mechanism to recover the signal z from the observation of y [19,22].

In order to utilize CS-based algorithms to solve the DoA estimation problem, the received signal model in (3) has to be transformed into a sparse representation model. The key idea is to divide the unambiguous DoA range into an equi-spaced angular grid containing potential DoAs of the sources. Let the ULA’s unambiguous DoA range from −π/2 to π/2 radians be scanned in the grid $\phi =\left[\phi_{1},\phi_{2},\ldots ,\phi_{N_{s}}\right]$, where $N_{s}\gg L$. Thus, the array steering matrix $A^{\prime }$ can be replaced by a virtual steering matrix A whose number of columns $N_{s}$ equals the cardinality of potential DoA set ϕ. As with other works [19,22,34] it is assumed that the source DoAs are included in ϕ. The virtual steering matrix A can be expressed as

(4) $$A=[a\left(\phi_{1}\right),a\left(\phi_{2}\right),\ldots ,a\left(\phi_{N_{s}}\right)].$$

Subsequently (3) can be rewritten as

(5) x=As+w,

where the vector s relates to the signal vector $s^{\prime }$ as  

(6) $$s_{n}=\left\{\begin{array}{ccc} s_{l}^{\prime }, & \phi_{n}=\theta_{l}, & n=1,2,\ldots ,N_{s}\\ & \; & l=1,2,\ldots ,L\\ 0, & \mathrm{otherwise} \end{array}.\right.$$

Here $s_{n}$ and $\phi_{n}$ are the *n*th elements of the sparse vector s and the vector ϕ, respectively. This representation allows the recovery of the *L*-sparse estimate $\hat{s}$ of s from measurement x by using A as the measurement or sensing matrix [34,47,48].

With $N_{s}\gg L$, most entries of s are zero and the DoA estimation problem is transformed into a standard CS problem of sparse recovery, where A and s are the measurement matrix and sparse vector, respectively. Given x and A, the problem of sparse signal reconstruction is to find $\hat{s}$, which can be cast as the minimization problem

(7) $$\hat{s_{i}}={\arg\min}_{s}{\;||s||}_{1}\;\;s.t.\;\;||x-{A\;s||}_{2}^{2}<\varepsilon ,$$

where ε is a small number bounded by the noise level [28]. Several techniques such as basis pursuit or iterative greedy algorithms can be used to solve (7) [22]. In this paper we also use a greedy algorithm, the OMP.

### 2.3. OMP Algorithm for DoA Estimation

The greedy methods for sparse signal recovery have faster convergence than $l_{1}$-minimization and they have been divided into two major categories namely the greedy pursuit methods and the thresholding-based methods [19,22]. Among the greedy pursuit methods, the OMP and compressive sampling matching pursuit (CoSaMP) are widely used, whereas, from the group of thresholding-based methods, iterative hard thresholding (IHT) is commonly used due to simplicity but at the cost of accuracy in signal recovery [22,23].

Now we give a brief overview of using the OMP algorithm for the DoA estimation problem under consideration. First, the OMP algorithm regards the output signal of the sparse array model x as the initial value of the residual error signal, $r_{0}$, and then correlates this residual signal with the column vectors $\gamma_{i};\;i=1,2,\ldots ,N_{s}$, of the sensing matrix A. Second, in each iteration, a column with the largest correlation is selected. Third, in each iteration, the components of the selected columns are removed from the residual error signal to obtain a new residual error signal $r_{i}$. The total number of iterations is the number of sources, i.e., *L*. The pseudocode of the standard OMP algorithm is given in Algorithm 1.

With $\hat{s}$ as the solution recovered by the OMP algorithm, the estimated angular spectrum can be expressed as

(8) $$p_{\phi }=\left[{\left|{\hat{s}}_{1}\right|}^{2},{\left|{\hat{s}}_{2}\right|}^{2},\ldots ,{\left|{\hat{s}}_{N_{s}}\right|}^{2}\right].$$

The locations of peaks observed in the plot of $p_{\phi }$ correspond to the estimated DoAs.
**Algorithm 1** Standard OMP Algorithm OMP-(Std)**Inputs**: x, A, *L***Output**: $\hat{s}$**Steps**:1. Initialize: residual $r_{0}\leftarrow x$, index set $\pi_{0}\leftarrow ⊘$,matrix $U_{0}\leftarrow \left[\;\right]$, iteration counter i←1**while** i≤L **do**    2. Find index $\omega_{i}$ of the column of A having maximum correlation with residual $r_{i-1}$   $\omega_{i}\leftarrow {\arg\max}_{j=1,2,\ldots ,N_{s}}\;|<r_{i-1},\;\gamma_{j}>|$   3. Update index set and matrix of selected columns   $\pi_{i}\leftarrow \pi_{i-1}\cup \;\omega_{i}$   $U_{i}\leftarrow \left[\;U_{i-1}\;\gamma_{\omega_{i}}\right]$   4. Solve ${\arg\min}_{s}\;||x-U_{i}\;s{\left|\right|}_{2}^{2}$ to estimate new sparse vector as   $\hat{s_{i}}\leftarrow {\left(U_{i}^{H}\;U_{i}\right)}^{-1}\;U_{i}^{H}\;x$   5. Update residual and iteration counter   $r_{i}\leftarrow x-U_{i}\;\hat{s_{i}}$   i←i+1**end while** **return** $\hat{s}\leftarrow \hat{s_{i}}$

### 2.4. CS-Based DoA Estimation for Multiple Snapshots

Considering the case where $T_{s}$ number of snapshots are measured; the single measurement vector model in (5) can be extended to the multiple measurement vector (MMV) case as

(9) X=AS+W,

where $X=\left[x\left(1\right),x\left(2\right),\ldots ,x\left(T_{s}\right)\right]\in C^{N\times T_{s}}$, $S=\left[s\left(1\right),s\left(2\right),\ldots ,s\left(T_{s}\right)\right]\in C^{N_{s}\times T_{s}}$, and $W=\left[w\left(1\right),w\left(2\right),\ldots ,w\left(T_{s}\right)\right]\in C^{N\times T_{s}}$. It is assumed that the true directions are time-invariant over the observation period of the $T_{s}$ snapshots. Therefore, only *L* rows of S are non-zero and S is said to have a jointly *L*-sparse structure [34]. As a result, the task of DoA estimation in the MMV case becomes a problem of determining the positions of non-zero elements in S.

A variety of algorithms exist that exploit the joint sparsity in different ways when S is not full rank [49]. Similar to the SMV case, two main approaches for solving MMV problems are convex optimization techniques and greedy methods. The counterpart of (7) in the MMV case can be expressed as

(10) $$\hat{S}={\arg\min}_{S}{\;||S||}_{2,1}\;\;s.t.\;\;||X-{A\;S||}_{2}^{2}\;<\;\varepsilon ,$$

where the $l_{p,q}$ norm of matrix S can be defined as [49]

(11) $${∥S∥}_{p,q}={\left(\sum_{i}{∥s_{i}∥}_{p}^{q}\right)}^{1/q},$$

with $s_{i}$ is the *i*th row of S for some p,q≥1.

The $l_{2,1}$-norm ensures that the number of the non-zero rows in $\hat{S}$ is the lowest. Once $\hat{S}$ is computed, the $l_{2}$-norms of the rows of $\hat{S}$ can be collected in a vector. By plotting the element-wise squares of this vector against the virtual angular grid indices $1,2,\ldots ,N_{s}$, the locations of peaks correspond to the estimated DoAs similar to (8).

Greedy methods, which are based on an extension of their SMV versions, are another popular approach to solve MMV problems. The simultaneous OMP (SOMP) algorithm [50] belongs to this category and is outlined in Algorithm 2. In SOMP, at each iteration, the column of A that simultaneously yields the best approximation to all of the residual vectors is selected. Particularly, at the *i*th iteration, we calculate an $N_{s}\times T_{s}$ matrix $C=A^{H}\;R_{i-1}$, where $R_{i-1}$ is the residual between the data matrix X and its approximation. In SOMP, we need to compute the $l_{p}$-norm for some p≥1 for each of the $N_{s}$ rows of C [Step (2) in Algorithm 2]. The row index corresponding to the largest $l_{p}$-norm is then selected to augment the index set. In the literature [51,52], p=1,p=2,andp=∞ have been used for solving the MMV problem. Except for Step 2, the remaining components in the SOMP algorithm are the same as those of the OMP algorithm described in Algorithm 1.

To summarize the above discussion on CS-based DoA estimation using the OMP algorithm, it can be noted that the sparse vector is estimated by the OMP algorithm and the estimated DoAs are the scanned angles that correspond to the *L* non-zero entries of the estimated sparse vector.
**Algorithm 2** Standard  Simultaneous OMP Algorithm SOMP-(Std)**Inputs**: X, A, *L***Output**: $\hat{S}$**Steps**:1. Initialize: residual $R_{0}\leftarrow X$, index set $\pi_{0}\leftarrow ⊘$,matrix $U_{0}\leftarrow \left[\;\right]$, iteration counter i←1**while** i≤L **do**    2. Find index $\omega_{i}$ of the column of A which best approximates all residuals   $\omega_{i}\leftarrow {\arg\max}_{j=1,2,\ldots ,N_{s}}\;||R_{i-1}^{H},\;\gamma_{j}{\left|\right|}_{p},\;p\geq 1$   3. Update index set and matrix of selected columns   $\pi_{i}\leftarrow \pi_{i-1}\cup \;\omega_{i}$   $U_{i}\leftarrow \left[\;U_{i-1}\;\gamma_{\omega_{i}}\right]$   4. Solve ${\arg\min}_{s}\;||X-U_{i}\;S{\left|\right|}_{2}^{2}$ to estimate new sparse matrix as   $P_{i}\leftarrow {\left(U_{i}^{H}\;U_{i}\right)}^{-1}\;U_{i}^{H}\;X$   5. Update residual and iteration counter   $R_{i}\leftarrow X-U_{i}\;\hat{P_{i}}$   i←i+1**end while** **return** $\hat{S}\leftarrow P_{i}$

## 3. Practical Error Models

This section explains the practical error models encountered in underwater deployment scenarios.

### 3.1. Faulty Sensors

Passive low frequency sonars are towed arrays deployed behind the ship and are used for detection, classification, localization, and tracking of underwater targets located at large distances [48]. In such towed arrays, the sensors are sealed in a tube-like structure to form an array and the sensors may get affected by a harsh natural environment such as electromagnetic interference, component aging, corrosion, or even physical damage that collectively increase the probability of failure of one or more of the sensors [30]. Furthermore, repair or replacement of sensors during the towed array operation is not an option. The beampattern of an array is its response in potential interference directions, for a particular steering direction. While the array gain of a large array is relatively insensitive to the failure of sensors in the array, in contrast the array beampattern is severely degraded due to increased side-lobe levels which significantly degrades the DoA estimation performance [29,40].

**Figure 2:**
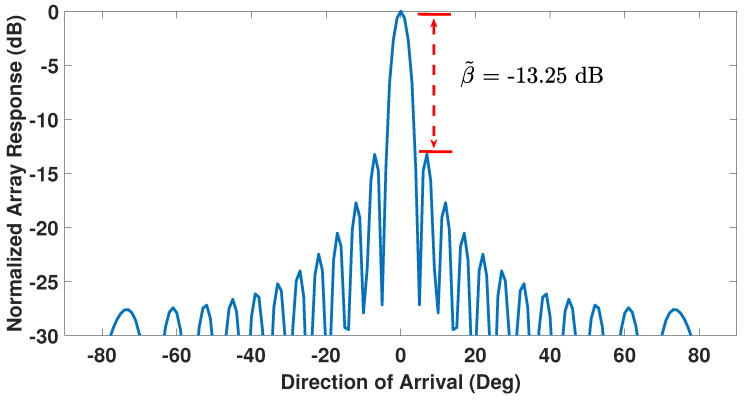
Beampattern of a 24-sensor ULA with equally powered and all-healthy sensors.

**Figure 3:**
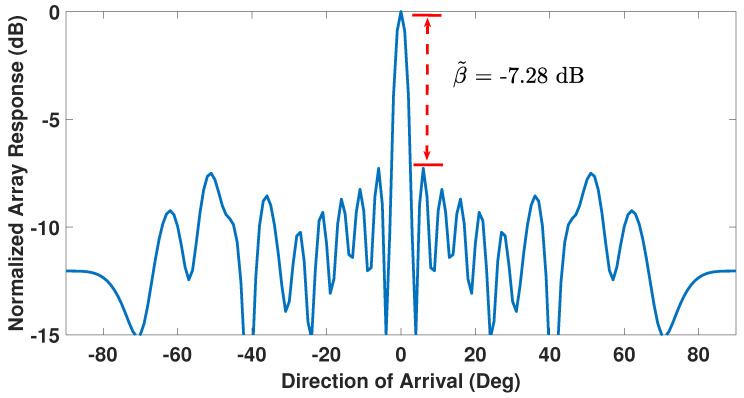
Beampattern of a 24-sensor ULA with six faulty sensors.

The beampattern of a ULA, with all its sensors functional and powered equally, exhibits a first side-lobe level of $\tilde{\beta }=-13.25$ dB relative to its main-lobe level [29,53] and is shown in Figure 2 for a 24-sensor ULA with half-wavelength spacing. With one or more faulty sensors, $\tilde{\beta }$ becomes a function of the number and positions of the faulty sensors, i.e., an increase in total number of faulty sensors or a reduction in distance between a faulty sensor and the array center causes $\tilde{\beta }$ to increase from its −13.25 dB reference value thereby undesirably increasing the first side-lobe level [29,40]. Figure 3 shows this behavior for the same 24-sensor ULA of Figure 2 but now with six failed sensors located at positions 5, 7, 10, 13, 16, and 20. Notice from Figure 3 that the side-lobe levels rise and the first side-lobe level is at −7.28 dB relative to the main lobe. This relative strength of the first side-lobe level can be defined in linear scale as $\beta =10^{\left(\tilde{\beta }/20\right)}$ [28].

A representative numeric example of side-lobe level increase with increasing number of faulty sensors in a 24-sensor ULA is given in Table 1.

It is pertinent to mention that sensor failures also disturb the placement and depth of beampattern nulls relative to their reference values in the healthy array which degrades the array’s interference rejection capability [53].

### 3.2. Low SNR

Radiated noise is the noise emitted by a vessel, and received by a hydrophone or an array of hydrophones at some distance from the vessel. Radiated noise is the source of signals for passive sonars which are designed to detect radiated noise against a background of ambient and self-noise. Over the decades an increase in marine traffic has raised the ambient noise levels, whereas potential military targets such as submarines, warships, and torpedoes use continuously evolving and sophisticated technologies to reduce their own radiation noise to avoid detection [38]. This results in typically low SNR values of the signals received from marine military targets which cannot be handled by passive techniques such as energy detection, and it has led to renewed research interest in acoustic array processing for target detection and classification in the underwater scenario [37]. The commonly used sensor array geometries include the linear, cylindrical, and spherical configurations. Mostly beamforming and other DoA estimation algorithms are applied on the data coming from sensor arrays and the display output is usually in the form of target bearing with respect to time and its amplitude [38].

In underwater sound propagation, signal intensity reduces because of spreading loss and absorption of acoustic energy by the propagating medium itself. Here SNR is the ratio of measured signal to background noise produced by everything else. It is the parameter that dictates the distance at which a target can be detected. In the underwater passive sonar scenario, the SNR at the sensor array can be written as [39,54]

(12) SNR=SL−TL−NL+AG,

where SL is the source level transmitted by the source, TL is the transmission loss, NL is the noise level, and AG is the array gain, with all these parameters expressed in dB.

The spectrum of signal radiated by different types of sonar targets is given in [39,54] in the form of a frequency (Hz) vs. spectrum level (dB) graph. Also the spectrum of ambient sea noise, often called the Wenz curve, is given in [39,54] in the form of a frequency (Hz) vs. spectrum level (dB) graph. Using these graphs, we can determine the value of SL and NL at a particular frequency. For example, the frequency range of interest for ships and submarines is between 100 and 500 Hz, depending upon their speed and depth. In addition, the TL is a range-dependent parameter. In the case of spherical spreading, the TL is calculated as 20logR [39,54]. Furthermore, the AG for a ULA can be calculated as 10logN and, with *N* = 24, the value of AG is 14 dB. Putting all these values in (12), the SNR at the sensor array, for two different target detection scenarios, is calculated in Table 2.

The SNR values in Table 2 are negative, which means that the received signal level is embedded in the background noise level [37,38]. Hence, sophisticated signal processing methods will be used to increase the SNR value. Without this processing the signal would be undetectable. Traditional array processing algorithms for DoA estimation have shown poor performance under unfavorable conditions. e.g., low SNR, limited measurement snapshots, and presence of coherent signal sources, whereas CS-based techniques can work with limited or even a single snapshot and can outperform the conventional methods under the aforementioned practical errors [9,55]. Keeping this in view, a lot of research has been going on in the CS framework towards using sparse reconstruction algorithms for DoA estimation [15,16].

Consider an underwater passive sensing scenario with a ULA having *N* sensors. The *L* far-field narrowband signal sources and the additive noise signals are assumed to be independently identically distributed (i.i.d.) Gaussian random processes with zero mean and variance $\sigma^{2}$, which can be varied to obtain various SNR values. In addition, each signal waveform is normalized such that $1/T_{s}\;\sum_{t=1}^{T_{s}}\;{\left|\left(s_{l}\left(t\right)\right)\right|}^{2}\;=\;\rho_{l}$, where l=1,2,…,L. Hence, the SNR can be defined in terms of signal and noise power as

(13) $$SNR_{dB}=10\log_{10}\left(\rho_{l}/\sigma^{2}\right).$$

Keeping the above in view, we consider three signal sources coming from different directions with signal powers 12 dB, 15 dB, and 10 dB, respectively. With noise power of 0 dB, the minimum SNR is 10 dB. Furthermore, we consider five signal sources coming from different directions with signal powers 20 dB, 20 dB, 25 dB, 25 dB, and 23 dB, respectively. With noise power of 35 dB, the minimum SNR is −15 dB. This definition of SNR has been used in Section 4, where different simulation scenarios are presented to illustrate the performance of the proposed MODE-OMP and MODE-SOMP algorithms in the presence of faulty sensors and low SNR, with single and multiple snapshots respectively.

### 3.3. Model-Order Estimation Errors

In general the array processing algorithms can address two basic issues from the array’s received composite signal, namely the model-order or source number detection and the directional estimates of the incoming signals. Some algorithms such as the Capon beamformer do not need the source-order information but have low DOA estimation accuracy and the maximum number of signals handled is at most *N*/2 [10]. Many other algorithms perform DoA estimation but require a priori knowledge of the source order. For example, the conventional subspace-based DoA estimation techniques require a priori knowledge of source order and they return inconsistent DoA estimates when an inaccurate estimate of source order is provided to them [11,12]. In this context, information theoretic approaches such as the AIC and MDL techniques have been widely used for model-order estimation [31,32]. These algorithms utilize the number of identical smallest eigenvalues of the received signal’s sample covariance matrix to estimate the number of signal sources *L*. However, in practical scenarios with low SNR and limited number of measurement snapshots, the AIC and MDL techniques both tend to estimate a wrong number of sources [31,32].

In recent years, CS-based sparse recovery algorithms such as the OMP and other iterative greedy algorithms have been proposed for DoA estimation [23]. These algorithms terminate their execution when some stopping rule is met which has been devised to meet acceptable performance levels [22]. While the algorithm’s reconstruction accuracy is typically measured in terms of energy of the residual error signal, the algorithm’s iteration count can be used as a measure of its complexity [19]. The standard OMP algorithm runs for a fixed number of iterations equal to the signal’s sparsity level or source order. This is an inherent drawback of the standard implementation as the source order may not be known a priori in practical underwater sensor array deployments [31,32]. Keeping all this in view, the standard greedy algorithms cannot be used for accurate DoA estimation unless the algorithm’s stopping criterion is modified to account for these errors, which is discussed in the next section.

## 4. Proposed MODE-OMP and MODE-SOMP Algorithms for Joint Model-Order and DoA Estimation

The CS-based iterative methods such as the OMP and SOMP algorithms use a priori information of the true source order *L* to terminate after *L* iterations with as many non-zero elements in the reconstructed sparse signal. However, if *L* is unknown which is the case in many practical sonar deployments, its over-estimation leads to components of noise being recovered as signal components. On the other hand, if the source order is under-estimated then some source DoAs may not be estimated by these reconstruction algorithms. With this view, modifications to the standard OMP and SOMP algorithms are proposed that do not require a priori knowledge of *L*. Here, the proposed algorithms are terminated by a modified stopping criterion that takes into account the array’s faulty sensors and low SNR conditions. The reconstruction algorithm’s successive residuals are evaluated in comparison with the maximum and the mean residue to terminate the algorithm when it is likely that noise components will be estimated as desired signal under unfavorable conditions of faulty sensors and low SNR. The faulty sensors can be included in the stopping criterion by incorporating the raised side-lobe levels into the threshold as [28] ${∥r_{i}∥}_{2}\geq \beta {∥r_{0}∥}_{2}$, where $r_{0}$ is the initial residual and $r_{i}$ is the *i*th iteration’s residual as previously discussed in Algorithm 1.

**Figure 4:**
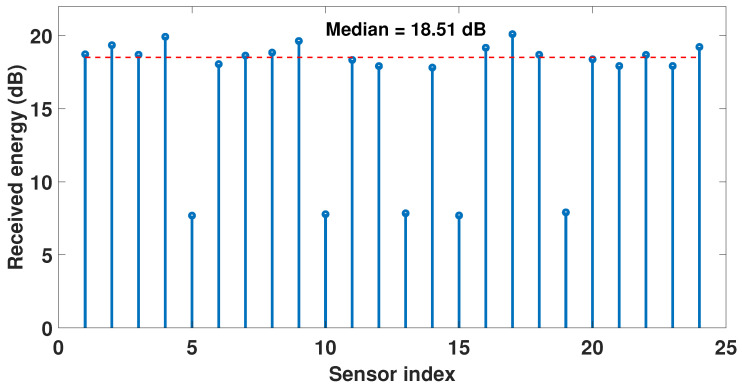
Identification of 5 faulty sensors in a 24-sensor ULA at index positions 5, 10, 13, 15, and 19. Other parameter values are SNR = 0 dB, $T_{s}=50$.

If the faulty sensor locations are not known then a computationally efficient scheme such as that proposed in [40] can be used for online detection of failed sensors with good accuracy and a relatively small number of snapshots compared with other failure detection schemes [56,57,58]. According to the adopted approach each sensor’s received signal energy is gauged by the signal variance estimated on that channel from several measurement snapshots. A faulty sensor will be identifiable by its minimal received energy and its variance significantly below the median variance across all sensor channels. Once all faulty sensors are identified then β can be calculated accordingly. Figure 4 shows a representative example of applying this method to identify five faulty sensors in a 24-sensor ULA. All those sensors whose received energies are below the median by 6 dB or more are identified as faulty. The first side-lobe level in this case is −7.8 dB relative to the mainlobe and the corresponding β=0.4 will be used in the modified stopping criterion to account for the faulty sensors.

The SNR conditions can be incorporated into the stopping criterion by noting that the mean residual energy for reconstruction relates with the variance of the identically distributed Gaussian sensor noise as [59]

(14) $$E\left[{∥r∥}_{2}^{2}\right]=N\sigma^{2},$$

where *N* is the number of sensors and $\sigma^{2}$ is the noise variance. Hence, to cater for SNR, the algorithm iterates as long as $∥r_{i}{∥}_{2}^{2}\geq N\sigma^{2}$.

The proposed stopping criterion is, therefore, a combination of the two stopping rules.

Stopping Rule 1—To handle faulty sensors.

(15) $$\left|\right|r_{i}{\left|\right|}_{2}\geq \beta \;||r_{0}{\left|\right|}_{2};\;i=i+1.$$

With an increase in the number of faulty array sensors, β increases and results in a larger threshold for $\left|\right|r_{i}{\left|\right|}_{2}$. This will reduce the number of algorithm iterations with more faulty sensors in the array.

Stopping Rule 2—To handle low SNR.

(16) $$\left|\right|r_{i}{\left|\right|}_{2}\geq \sqrt{N}\;\sigma ;\;i=i+1.$$

For given signal power, increasing σ corresponds to reducing SNR and results in a larger threshold for $\left|\right|r_{i}{\left|\right|}_{2}$. This will reduce the number of algorithm iterations for low SNR conditions so that noise artifacts are not erroneously detected as signal components.

The joint application of these two stopping rules can be stated as

(17) $$\left|\right|r_{i}{\left|\right|}_{2}\geq \max\;(\beta \;||r_{0}{\left|\right|}_{2},\;\sqrt{N}\;\sigma ),$$

where the number of algorithm iterations and the cardinality of the recovered sparse signal is determined by the maximum of the two thresholds. For a 24- or 48-sensor ULA and low SNR of −20 dB, $\beta ∥r_{0}{∥}_{2}$ is observed to be greater than $\sqrt{N}\sigma$. On the other hand, with the number of sensors exceeding 100 and lower SNR of −25 dB or −30 dB, the $\sqrt{N}\sigma$ factor is observed to be larger than $\beta ∥r_{0}{∥}_{2}$. However, the proposed stopping criterion can successfully handle both situations by using the β and σ parameters.

The DoA estimation based on the proposed MODE-OMP and MODE-SOMP algorithms is given in Algorithms 3 and 4, respectively. It can be noted that the sparse vector, in both algorithms, is estimated using the proposed stopping criterion in (17), and the DoAs correspond to the distinct non-zero entries in the estimated sparse vector.
**Algorithm 3** MODE-OMP  Algorithm**Inputs**: x, A, β, $\sigma^{2}$**Outputs**: $\hat{s}$**Steps**:1. Initialize: residual $r_{0}\leftarrow x$, $\psi_{0}$$\leftarrow \left|\right|r_{0}{\left|\right|}_{2}$,index set $\pi_{0}\leftarrow ⊘$, matrix $U_{0}\leftarrow \left[\;\right]$, N=length(x),iteration counter i←0**while** $\psi_{i}\geq \max\left(\beta \psi_{0},\sqrt{N}\sigma \right)$ **do**    i←i+1   2. Find index $\omega_{i}$ of the column of A having maximum correlation with residual $r_{i-1}$   $\omega_{i}\leftarrow {\arg\max}_{j=1,2,\ldots ,N_{s}}\;|<r_{i-1},\;\gamma_{j}>|$   3. Update index set and matrix of selected columns   $\pi_{i}\leftarrow \pi_{i-1}\cup \;\omega_{i}$   $U_{i}\leftarrow \left[\;U_{i-1}\;\gamma_{\omega_{i}}\right]$   4. Solve ${\arg\min}_{s}\;||x-U_{i}\;s{\left|\right|}_{2}^{2}$ to estimate new sparse vector as   $\hat{s_{i}}\leftarrow {\left(U_{i}^{H}\;U_{i}\right)}^{-1}\;U_{i}^{H}\;x$   5. Update residual   $r_{i}\leftarrow x-U_{i}\;\hat{s_{i}}$   $\psi_{i}\leftarrow \left|\right|r_{i}{\left|\right|}_{2}$**end while** **return** $\hat{s}\leftarrow \hat{s_{i}}$

**Algorithm 4** MODE-SOMP Algorithm
**Inputs**: X, A, β, $\sigma^{2}$**Outputs**: $\hat{S}$**Steps**:1. Initialize: residual $R_{0}\leftarrow X$, $\psi_{0}=\left|\right|R_{0}{\left|\right|}_{2}$, index set $\pi_{0}\leftarrow ⊘$, matrix $U_{0}\leftarrow \left[\;\right]$, N=length(x), iteration counter i←0**while** $\psi_{i}\geq \max\left(\beta \psi_{0},\sqrt{N}\sigma \right)$ **do**    i←i+1   2. Find index $\omega_{i}$ of the column of A which best approximates all residuals   $\omega_{i}\leftarrow {\arg\max}_{j=1,2,\ldots ,N_{s}}\;||R_{i-1}^{H},\;\gamma_{j}{\left|\right|}_{p},\;p\geq 1$   3. Update index set and matrix of selected columns   $\pi_{i}\leftarrow \pi_{i-1}\cup \;\omega_{i}$   $U_{i}\leftarrow \left[\;U_{i-1}\;\gamma_{\omega_{i}}\right]$   4. Solve ${\arg\min}_{s}\;||X-U_{i}\;S{\left|\right|}_{2}^{2}$ to estimate new sparse matrix as   $P_{i}\leftarrow {\left(U_{i}^{H}\;U_{i}\right)}^{-1}\;U_{i}^{H}\;X$   5. Update residual   $R_{i}\leftarrow X-U_{i}\;P_{i}$   $\psi_{i}=\left|\right|R_{i}{\left|\right|}_{2}$**end while** **return** $\hat{S}\leftarrow P_{i}$


## 5. Simulation Results

In this section, some numerical results are provided to show the performance gains of the proposed MODE-OMP and MODE-SOMP algorithms in relation to the standard OMP and SOMP algorithms, the MFOCUSS algorithm, and the IAA sparse estimator. A 24-sensor ULA with half-wavelength element spacing is considered and in total 181 scanning angles, uniformly distributed over the ULA’s unambiguous angular range −π/2 to π/2 radians, are considered in the numerical evaluations. The sensor noise is modeled as spatially and temporally white and identically Gaussian distributed. The statistical performance evaluation is conducted using 1000 Monte Carlo trials for each of the four distinct scenarios considered in the numerical evaluations. In scenarios 1, 2, and 3 the number and array index of the faulty sensors are known a priori, whereas these parameters are estimated in scenario 4. Furthermore, scenarios 1, 2, and 4 consider incoming signals with equal powers without loss of generality, whereas the case of un-equal signal powers is treated in scenario 3 for completeness.

The comparative performance of the proposed MODE-OMP and MODE-SOMP algorithms is given in relation to that of the standard OMP and SOMP implementations with source order known and then estimated, as well as the IAA algorithm and the MFOCUSS algorithm with its parameter values suitably set as *p* = 0.8, maximum iteration number set to 2*N* = 48, and the stopping threshold set to $10^{-5}$ [60,61].

The comparative recovery performance of the aforementioned algorithms is analyzed in terms of the RMSE of DoA estimates defined as [34,62,63]

(18) $$\mathrm{RMSE}\left(\theta \right)=\sqrt{\frac{1}{\xi \;L}\sum_{i=1}^{\xi }\sum_{l=1}^{L}\left({\left|{\hat{\theta }}_{i,l}-\theta_{l}\right|}^{2}\right)},$$

where ξ=1000 is the total number of Monte Carlo trials and ${\hat{\theta }}_{i,l}$ is the estimated result of $\theta_{l}$ in the *i*th Monte Carlo trial.

Furthermore, the probability of target resolution is defined over 1000 Monte Carlo runs and is computed by dividing the number of successful target resolutions with the number of Monte Carlo runs. Lastly, the median iteration count of all the considered algorithms over 1000 successive Monte Carlo runs is also compared.

### 5.1. SCENARIO 1: Six Faulty Sensors at Positions 3, 5, 9, 12, 15, and 18

**Figure 5:**
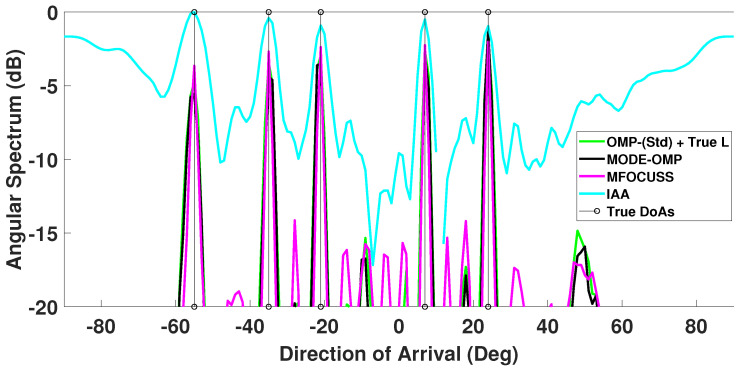
Comparison of DoA estimates by proposed MODE-OMP, OMP-(Std) using *L* known, MFOCUSS, and IAA algorithms. The true L=5 DoAs at −55${}^{\circ }$, −35${}^{\circ }$, −21${}^{\circ }$, 7${}^{\circ }$, 24${}^{\circ }$ are plotted for reference. Other parameter values are SNR = −15 dB, $T_{s}=1$.

In this scenario, five equal power signals are received at DoAs $-55^{\circ }$, $-35^{\circ }$, $-21^{\circ }$, $7^{\circ }$, and $24^{\circ }$, respectively. There are six faulty sensors at known ULA indices 3, 5, 9, 12, 15, and 18. The corresponding angular spectrum is plotted in Figure 5. For the given faulty sensor positions, the first side-lobe level is −7.6 dB relative to the main-lobe level and the corresponding β is 0.45 as calculated by the approach discussed previously in Section 3. From Figure 5 it can be observed that for the single-snapshot case, i.e., $T_{s}=1$, and a low SNR of −15 dB, the proposed MODE-OMP algorithm has identical performance to the standard OMP with known source order *L*.

**Figure 6:**
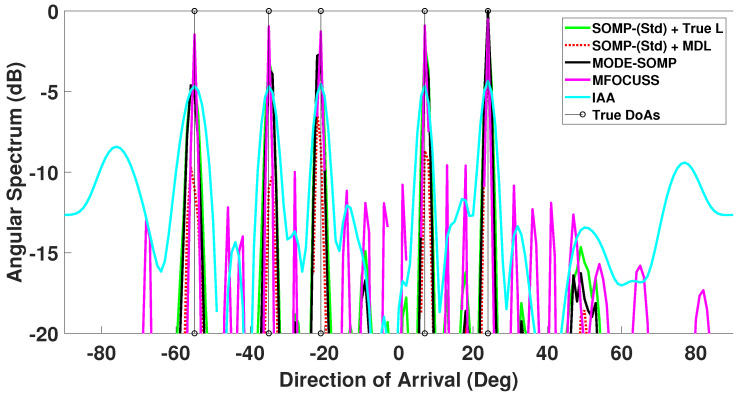
Comparison of DoA estimates by proposed MODE-SOMP algorithm, SOMP-(Std) using *L* known and *L* estimated, MFOCUSS, and IAA algorithms. The true L=5 DoAs at −55${}^{\circ }$, −35${}^{\circ }$, −21${}^{\circ }$, 7${}^{\circ }$, 24${}^{\circ }$ are plotted for reference. Other parameter values are SNR = −15 dB, $T_{s}=25$.

In Figure 6 the estimated angular spectrum for the multi-snapshot case with $T_{s}=25$ is shown. It can be seen from this figure that the proposed MODE-SOMP performs identically to the standard SOMP algorithm that uses a priori known *L*. For comparison, the angular spectrum of the standard SOMP algorithm with *L* estimated by MDL technique is also plotted. It can be observed from the figure that the spectral peaks corresponding to true DoAs with the MDL approach are not as well-pronounced as those of the MODE-SOMP and standard SOMP with known *L*. This follows from inaccuracies in MDL estimates at low SNR [31,32]. Furthermore, the MDL technique has another drawback that it requires number of snapshots ≥ number of array sensors [31,32] and so it cannot be used in the single-snapshot case.

In addition, Figure 5 and Figure 6 show the angular spectrum of the MFOCUSS and IAA techniques for single- and multiple-snapshot cases, respectively. The spatial spectrum of the MFOCUSS algorithm in [64] shows many smaller spectral peaks for a 16-sensor ULA, receiving two far-field signal sources, SNR set to 5 dB, $T_{s}$ = 100 snapshots and grid interval of $1^{\circ }$. In case of low SNR, i.e., SNR = −15 dB, Figure 6 shows many raised spectral peaks in the entire spectrum of the MFOCUSS algorithm. The IAA method provides accurate DoA estimates in the high SNR region (SNR > 0 dB) [24,25]. In the low SNR regime (SNR < 0 dB), the IAA angular spectrum shows performance degradation as shown in Figure 5, where the IAA output is shifted upwards indicating high background noise and high secondary lobes in the end-fire regions indicating false peaks as seen in Figure 6.

**Figure 7:**
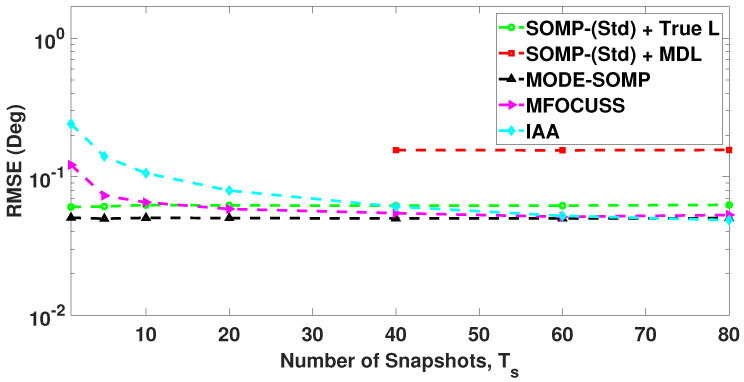
RMSE of DoA estimates plotted as a function of number of snapshots. The true L=5 DoAs are at −55${}^{\circ }$, −35${}^{\circ }$, −21${}^{\circ }$, 7${}^{\circ }$, 24${}^{\circ }$. Other parameter value is SNR = −15 dB.

In Figure 7, we compare the RMSE plots of DoA estimates by the standard and proposed algorithms for different numbers of snapshots. In this case the RMSE is calculated with a total of 1000 Monte Carlo trials. Figure 7 shows the RMSE plot of standard SOMP with estimated *L* for 40, 60, and 80 snapshots. On the other hand, Figure 7 depicts the RMSE plots of standard SOMP with true *L*, proposed MODE-SOMP, MFOCUSS, and IAA algorithms for 1, 5, 10, 20, 40, 60, and 80 snapshots. In the case of a single snapshot, i.e., $T_{s}=1$, the SOMP algorithm is reduced to the OMP algorithm as discussed previously in Section 2. Figure 7 clearly shows that the proposed algorithms achieve the lowest RMSE across the snapshots compared to all the aforementioned techniques.

**Figure 8:**
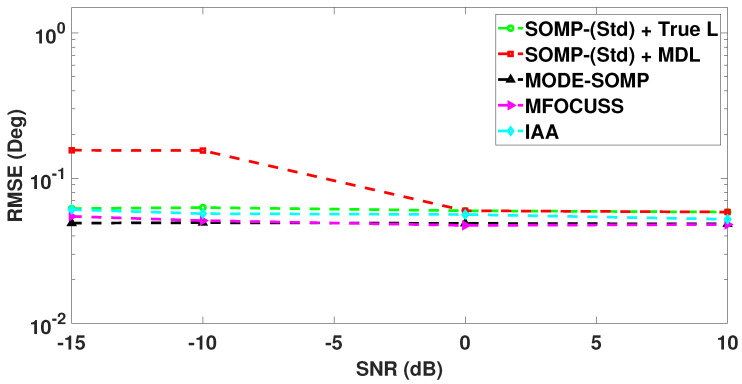
RMSE of DoA estimates plotted as a function of received SNR. The true L=5 DoAs are at −55${}^{\circ }$, −35${}^{\circ }$, −21${}^{\circ }$, 7${}^{\circ }$, 24${}^{\circ }$. Other parameter value is $T_{s}=40$.

In Figure 8, we analyze the RMSE plots of DoA estimates by the standard and proposed algorithms against different values of received SNR. The number of snapshots is set to $T_{s}=40$. With the results shown in Figure 8, it can be seen that the proposed algorithms achieve the lowest RMSE in the low SNR region (SNR < 0 dB) and similar RMSE in the high SNR region (SNR ≥ 0 dB), without asking for the model-order information, when compared to all the other techniques under consideration.

Table 3 and Table 4 compare the standard and proposed algorithms in terms of median iteration count over 1000 trials for single- and multi-snapshot cases, respectively. The standard OMP and SOMP algorithms run for a fixed number of iterations, i.e., true source order *L*. For low SNR values, i.e., SNR < 0 dB, the standard SOMP algorithm runs for a smaller number of iterations as shown in Table 4, due to wrong source order estimation by the MDL technique in the low SNR condition. This effect of wrong source order estimation by MDL can be seen in Figure 6 for source DoAs at $-35^{\circ }$ and $-21^{\circ }$, where the spectral peaks obtained through the MDL approach are not aligned with the true DoAs. On the other hand, for high SNR, i.e., SNR ≥ 0 dB, in comparison to standard OMP and SOMP algorithms with true source order *L*, the proposed MODE-OMP and MODE-SOMP algorithms achieve similar performance for single- and multi-snapshot cases, respectively, without requiring prior information about the model order.

Table 3 and Table 4 show that for any given scenario, i.e., fixed number of snapshots, faulty sensors, and low SNR, the MODE-OMP and MODE-SOMP algorithms provide accurate DoA estimation while requiring number of iterations equal to the standard OMP, standard SOMP, and IAA techniques. It is pertinent to mention here that the proposed algorithms leverage the additional information of β and σ in their stopping criteria to jointly handle the unknown source order, faulty array sensors, and low SNR conditions.

**Figure 9:**
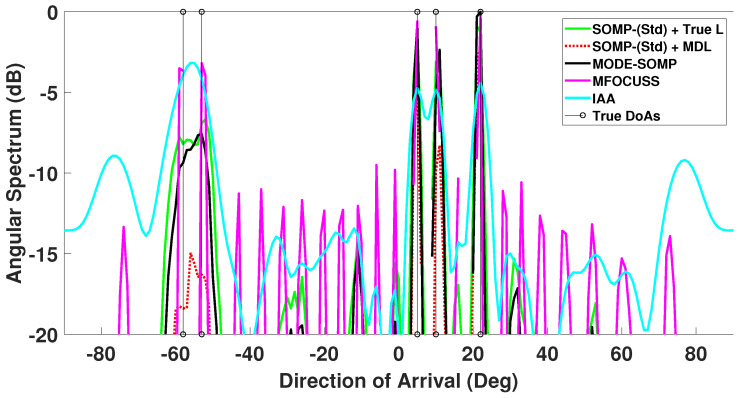
Comparison of DoA estimates by proposed MODE-SOMP algorithm, SOMP-(Std) using *L* known and *L* estimated, MFOCUSS, and IAA algorithms. The true L=5 DoAs at −58${}^{\circ }$, −53${}^{\circ }$, 5${}^{\circ }$, 10${}^{\circ }$, 22${}^{\circ }$ are plotted for reference. Other parameter values are SNR = −15 dB, $T_{s}=25$.

In Figure 9, the angular spectrum is shown in which all the algorithms are able to distinguish the signal sources as long as their angular separation is ≥the Rayleigh limit or approximate beamwidth relative to array boresight. An approximation of beamwidth for a ULA with half-wavelength sensor spacing comes out to be 100/*N* (deg) relative to array boresight [65]. The beamwidth widens as the beam is scanned away from the boresight. The approximate beamwidth is $4^{\circ }$ for a 24-sensor ULA. It is well known that the conventional OMP and SOMP algorithms cannot distinguish between two targets, when the angle difference between them is lower than the Rayleigh limit [34,65]. In Figure 9, it can be seen that all the standard and proposed algorithms are able to distinguish the signal sources as long as their DoA difference is ≥$4^{\circ }$ relative to array boresight, i.e., $5^{\circ }$ and $10^{\circ }$, respectively. On the other hand, when the two DoAs are located away from the array boresight, i.e., $-58^{\circ }$ and $-53^{\circ }$ in this scenario, all the standard and proposed algorithms failed to distinguish the two signal sources as shown in Figure 9. Hence, the results obtained are consistent with theory and show that the angular resolution of standard and proposed OMP and SOMP algorithms is the Rayleigh limit proportional to the array aperture N×d [34].

### 5.2. SCENARIO 2: Ten Faulty Sensors at Positions 3, 5, 7, 9, 12, 15, 16, 18, 22, and 23

In this scenario, five equal power signals are received at DoAs $-27^{\circ }$, $-9^{\circ }$, $5^{\circ }$, $17^{\circ }$, and $32^{\circ }$. There are ten faulty sensors at known ULA indices 3, 5, 7, 9, 12, 15, 16, 18, 22, and 23. For the given faulty sensor positions, the first side-lobe level is −5.7 dB relative to the main lobe level and the corresponding β is 0.56 as calculated by the approach discussed previously in Section 3.

**Figure 10:**
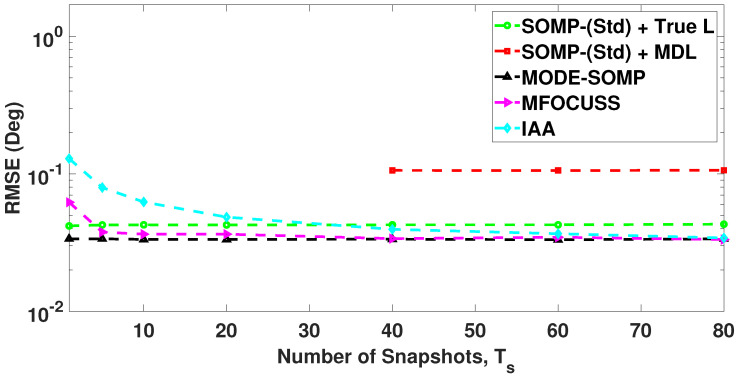
RMSE of DoA estimates plotted as a function of number of snapshots. The true L=5 DoAs are at −27${}^{\circ }$, −9${}^{\circ }$, 5${}^{\circ }$, 17${}^{\circ }$, 32${}^{\circ }$. Other parameter value is SNR = −15 dB.

In Figure 10, we compare the RMSE plots of DoA estimates by the standard and proposed algorithms with different numbers of snapshots, computed over 1000 Monte Carlo trials. Figure 10 shows the RMSE plot of standard SOMP with estimated *L* for 40, 60, and 80 snapshots. On the other hand, Figure 10 depicts the RMSE plots of standard SOMP with true *L*, proposed MODE-SOMP, MFOCUSS, and IAA algorithms for 1, 5, 10, 20, 40, 60, and 80 snapshots. In the case of a single snapshot, i.e., $T_{s}=1$, the SOMP algorithm is reduced to the OMP algorithm as discussed previously in Section 2. Figure 10 clearly shows that the proposed algorithms achieve the lowest RMSE across the snapshots compared to all the aforementioned techniques.

**Figure 11:**
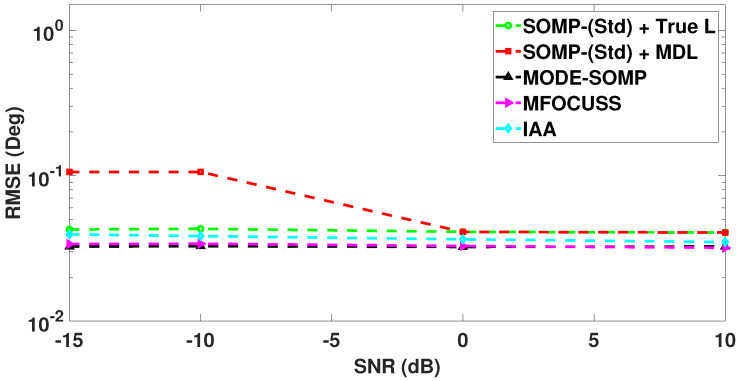
RMSE of DoA estimates plotted as a function of received SNR. The true L=5 DoAs are at −27${}^{\circ }$, −9${}^{\circ }$, 5${}^{\circ }$, 17${}^{\circ }$, 32${}^{\circ }$. Other parameter value is $T_{s}=40$.

In Figure 11, we analyze the RMSE plots of DoA estimates by the standard and proposed algorithms against different values of received SNR with $T_{s}=40$ snapshots. With the results shown in Figure 11, it can be seen that the proposed algorithms achieve the lowest RMSE in the low SNR region (SNR < 0 dB) and similar RMSE in the high SNR region (SNR ≥ 0 dB), without a priori knowledge of model order, when compared to all the other techniques under consideration.

The results in terms of median iteration count over 1000 trials for the single-snapshot case, i.e., $T_{s}=1$, show a similar trend to that described previously in Section 5.1 with Table 3. Table 5 compares the standard and proposed algorithms in terms of median iteration count over 1000 trials for the multi-snapshots case. In the low SNR condition (SNR < 0 dB), the standard SOMP algorithm runs for a smaller number of iterations as shown in Table 5, due to wrong source order estimation by the MDL technique. On the other hand for high SNR (SNR ≥ 0 dB), in comparison to standard OMP and SOMP algorithms with true source order *L*, the proposed MODE-OMP and MODE-SOMP algorithms achieve similar performance for single- and multi-snapshot cases, respectively.

Table 5 shows that for any given scenario, i.e., fixed number of snapshots, faulty sensors, and low SNR, the MODE-OMP and MODE-SOMP algorithms provide accurate DoA estimation while requiring a smaller number of iterations compared to the standard OMP, standard SOMP, and IAA techniques. It is pertinent to mention here that the proposed algorithms leverage the additional information of β and σ in their stopping criteria to jointly handle the unknown source order, faulty array sensors, and low SNR conditions.

### 5.3. SCENARIO 3: Six Faulty Sensors at Positions 3, 4, 11, 13, 21, and 24, and Unequal Signal Strengths

In this scenario, five signals with unequal power are received at DoAs $-18^{\circ }$, $-10^{\circ }$, $0^{\circ }$, $9^{\circ }$, and $17^{\circ }$, respectively. First, we consider signal sources with power of 12 dB, 15 dB, 10 dB, 10 dB, and 15 dB. The noise power is 0 dB, which results in a minimum SNR of 10 dB. Second, we consider signal sources with power of 20 dB, 20 dB, 25 dB, 25 dB, and 23 dB. The noise power is 35 dB, which results in a minimum SNR of −15 dB. There are six faulty sensors at known ULA indices 3, 4, 11, 13, 21, and 24. For the given faulty sensor positions, the first side-lobe level is −8.8 dB relative to the main-lobe level and the corresponding β is 0.39 as calculated by the approach discussed previously in Section 3.

**Figure 12:**
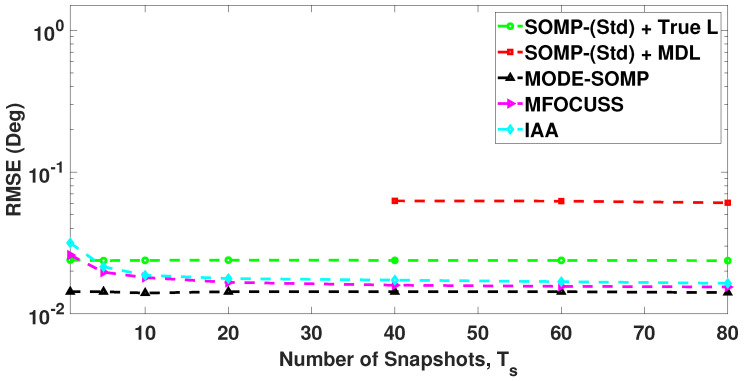
RMSE of DoA estimates plotted as a function of number of snapshots. The true L=5 DoAs are at −18${}^{\circ }$, −10${}^{\circ }$, 0${}^{\circ }$, 9${}^{\circ }$, 17${}^{\circ }$. Other parameter value is SNR = −15 dB.

In Figure 12, we compare the RMSE plots of DoA estimates by the standard and proposed algorithms with different numbers of snapshots, computed over 1000 Monte Carlo trials. Figure 12 shows the RMSE plot of standard SOMP with estimated *L* for 40, 60, and 80 snapshots. On the other hand, Figure 12 depicts the RMSE plots of standard SOMP with true *L*, proposed MODE-SOMP, MFOCUSS, and IAA algorithms for 1, 5, 10, 20, 40, 60, and 80 snapshots. Figure 12 clearly shows that the proposed algorithms achieve the lowest RMSE across the snapshots compared to all the aforementioned techniques.

**Figure 13:**
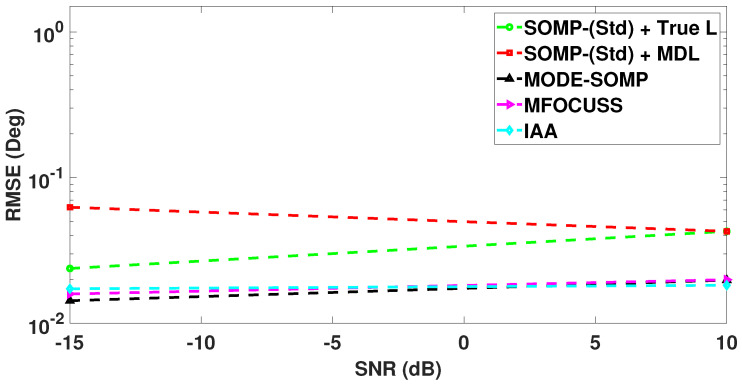
RMSE of DoA estimates plotted as a function of received SNR. The true L=5 DoAs are at −18${}^{\circ }$, −10${}^{\circ }$, 0${}^{\circ }$, 9${}^{\circ }$, 17${}^{\circ }$. Other parameter value is $T_{s}=40$.

In Figure 13, we analyze the RMSE plots of DoA estimates by the standard and proposed algorithms against two received SNR values, i.e., −15 and 10 dB with $T_{s}=40$ snapshots. Figure 13 shows that the proposed algorithms achieve the lowest RMSE at both SNR values, when compared to all the other techniques under consideration.

The results in terms of median iteration count over 1000 trials for the single-snapshot case, i.e., $T_{s}=1$, show a similar trend to that described previously in Section 5.1 with Table 3. Table 6 compares the standard and proposed algorithms in terms of median iteration count over 1000 trials for the multi-snapshot case. In the low SNR condition (SNR < 0 dB), the standard SOMP algorithm runs for a smaller number of iterations as shown in Table 6, due to wrong source order estimation by the MDL technique. On the other hand for high SNR (SNR ≥ 0 dB), in comparison to standard OMP and SOMP algorithms with true source order *L*, the proposed MODE-OMP and MODE-SOMP algorithms achieve similar performance for single- and multi-snapshots cases, respectively.

Table 6 shows that for any given scenario, i.e., fixed number of snapshots, faulty sensors, and low SNR, the MODE-OMP and MODE-SOMP algorithms provide accurate DoA estimation while requiring number of iterations equal to the standard OMP, standard SOMP, and IAA techniques. It is pertinent to mention here that the proposed algorithms leverage the additional information of β and σ in their stopping criteria to jointly handle the unknown source order, faulty array sensors, and low SNR conditions.

### 5.4. SCENARIO 4: Unknown Number and Positions of Faulty Sensors

In this scenario, there are six faulty sensors located at ULA indices 8, 11, 13, 15, 17, and 20. However, the number of these faulty sensors and their array positions are not known a priori and so they must be estimated to subsequently compute the β parameter by using the approach previously discussed in Section 3. It is pertinent to mention here that the true value of the estimated β that corresponds to actual locations of the faulty sensors is $\beta_{\mathrm{true}}=0.48$. Furthermore, this simulation scenario assumes five equal power signals impinging on the ULA with DoAs −27${}^{\circ }$, −18${}^{\circ }$, 2${}^{\circ }$, 15${}^{\circ }$, and 24${}^{\circ }$.

**Figure 14:**
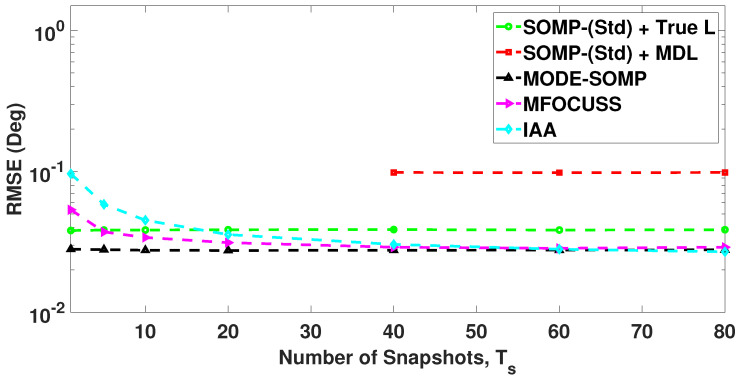
RMSE of DoA estimates plotted as a function of number of snapshots. The true L=5 DoAs are at −27${}^{\circ }$, −18${}^{\circ }$, 2${}^{\circ }$, 15${}^{\circ }$, 24${}^{\circ }$. Other parameter value is SNR = −15 dB.

In Figure 14, the RMSE of DoA estimates returned by the proposed MODE-SOMP algorithm is plotted as a function of the number of snapshots. For comparison, the RMSE of DoA estimates given by the standard SOMP with a priori known *L* and the standard SOMP with *L* estimated by the MDL technique are also shown. In the single-snapshot case, $T_{s}=1$, the SOMP algorithm is reduced to the OMP algorithm as previously discussed in Section 2. Figure 14 clearly shows that the proposed algorithms achieve a lower RMSE for any number of snapshots compared to all the aforementioned techniques.

**Figure 15:**
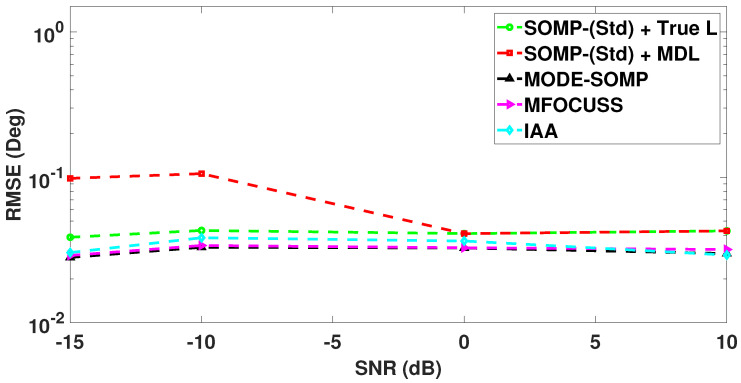
RMSE of DoA estimates plotted as a function of received SNR. The true L=5 DoAs are at −27${}^{\circ }$, −18${}^{\circ }$, 2${}^{\circ }$, 15${}^{\circ }$, 24${}^{\circ }$. Other parameter value is $T_{s}=40$.

In Figure 15, we demonstrate the RMSE of DoA estimates as a function of the received SNR for a fixed number of snapshots, $T_{s}=40$. Figure 15 clearly shows that the proposed MODE-SOMP algorithm achieves the lowest RMSE consistently and in fact its performance gain relative to the other schemes gradually increases with decreasing SNR values (SNR < 0 dB).

The results in terms of median iteration count over 1000 trials for the single-snapshot case, i.e., $T_{s}=1$, show a similar trend to that described previously in Section 5.1 with Table 3. Table 7 compares the standard and proposed algorithms in terms of median iteration count over 1000 Monte Carlo trials for the multi-snapshot case. When source order *L* is known a priori, the standard SOMP algorithm runs for a fixed number of iterations equal to *L* as shown in Table 7. However, when *L* has to be estimated by using MDL, then at the low SNR values, e.g., SNR = −15 dB, the standard SOMP runs erroneously for a smaller number of iterations due to the wrong estimate of source order by the MDL technique under low SNR conditions as shown in Table 7. On the other hand, in the high SNR regime (SNR ≥ 0 dB) the proposed MODE-OMP and MODE-SOMP algorithms have identical performance to the standard algorithms with known *L* in the single- and multi-snapshot cases, respectively.

Table 7 shows that for any given scenario, i.e., fixed number of snapshots, faulty sensors, and low SNR, the MODE-OMP and MODE-SOMP algorithms provide accurate DoA estimation while requiring a similar order of iterations as those required by the standard OMP, standard SOMP, and IAA techniques. It is pertinent to mention here that the proposed algorithms leverage the additional information of β and σ in their stopping criteria to jointly handle the unknown source order, faulty array sensors, and low SNR conditions.

**Figure 16:**
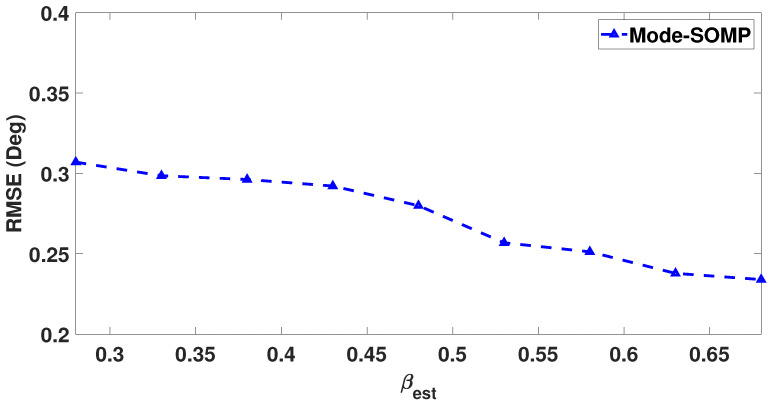
RMSE of DoA estimates plotted as a function of $\beta_{\mathrm{est}}$ for proposed MODE-SOMP algorithm. Other parameter values are SNR = −15 dB, $T_{s}=25$.

In this scenario, it is pertinent to mention that the estimated parameter $\beta_{\mathrm{est}}$ may be different from its true value $\beta_{\mathrm{true}}$. So the effect of erroneous $\beta_{\mathrm{est}}$ on RMSE(θ) needs to analyzed. Figure 16 exhibits the RMSE(θ) plotted as a function of $\beta_{\mathrm{est}}$ fluctuations around its true value $\beta_{\mathrm{true}}$ = 0.48. The figure shows that the variation in RMSE(θ) is negligible over the explored range of $\beta_{\mathrm{est}}$ values.

**Figure 17:**
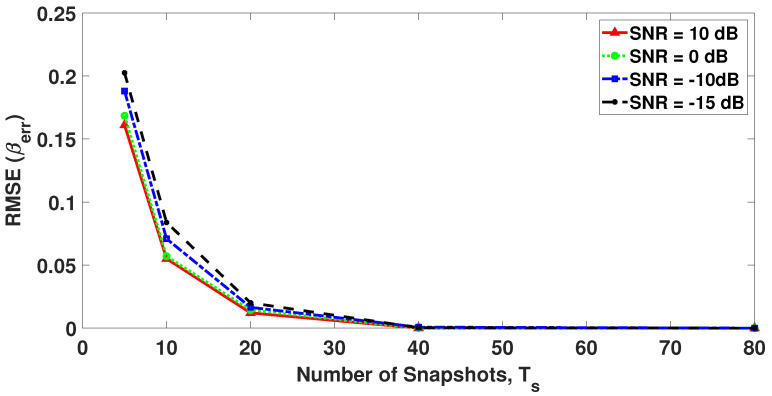
RMSE ($\beta_{\mathrm{err}}$) plotted as a function of number of snapshots for 6 faulty sensors. Other parameter values are received SNR = −15, −10, 0, and 10 dB.

To further analyze the effect of erroneous $\beta_{\mathrm{est}}$ on DoA estimates, another RMSE indicator in terms of $\beta_{\mathrm{est}}$ and $\beta_{\mathrm{true}}$ is formulated as

(19) $$\mathrm{RMSE}\left(\beta_{\mathrm{err}}\right)=\sqrt{\frac{1}{\xi }\sum_{i=1}^{\xi }\left({\left|\beta_{\mathrm{est},i}-\beta_{\mathrm{true}}\right|}^{2}\right)},$$

where ξ=1000 is the total number of Monte Carlo trials and $\beta_{\mathrm{est},i}$ is the estimated value of $\beta_{\mathrm{est}}$ in the *i*th Monte Carlo trial. Figure 17 shows the RMSE($\beta_{\mathrm{err}}$) as a function of the number of snapshots at different SNR values. Figure 17 shows that, for $T_{s}\geq 10$ snapshots, the value of RMSE($\beta_{\mathrm{err}}$) is very small and it starts to approach zero for $T_{s}\geq 20$, i.e., $\beta_{\mathrm{est}}$ approaches $\beta_{\mathrm{true}}$. These results highlight the significance of using the proposed MODE-SOMP algorithm for DoA estimation in the multi-snapshot case as it is shown to achieve similar performance to the standard SOMP algorithm with known *L* without requiring prior information of the model order. Also, if β is required to be estimated for MODE-SOMP then the required number of snapshots for its estimation will be typically smaller than the number of snapshots required to estimate the source order required by the standard SOMP algorithm that relies on source order estimation techniques such as the MDL.

### 5.5. Probability of Target Resolution

It is well known that the standard OMP and SOMP algorithms cannot distinguish between two targets, when the angle difference between them is lower than the Rayleigh limit [34,65]. In Figure 9, it is shown that all the standard and proposed algorithms are able to distinguish the signal sources as long as their DoA difference is ≥$4^{\circ }$ relative to the array boresight. Keeping this in view, we consider two sources positioned at $\theta_{1}=10^{\circ }$ and $\theta_{2}=14^{\circ }$ with respect to the array axis and near the array boresight, while using the parameters employed for scenario 2, i.e., the 24-sensor ULA has ten faulty sensors at known indices 3, 5, 7, 9, 12, 15, 16, 18, 22, and 23. For the given faulty sensor positions, the first side-lobe level is −5.7 dB relative to the main-lobe level and the corresponding β is 0.56 as calculated by the approach discussed previously in Section 3. The estimated DoAs are denoted as $\hat{\theta_{1}}$ and $\hat{\theta_{2}}$, respectively. The two targets are said to be successfully resolved when the following equation is satisfied [66,67,68]

(20) $$\left|\theta_{i}-\hat{\theta_{i}}\right|\leq \left(\theta_{2}-\theta_{1}\right)/\;2,\;i=1,2.$$

**Figure 18:**
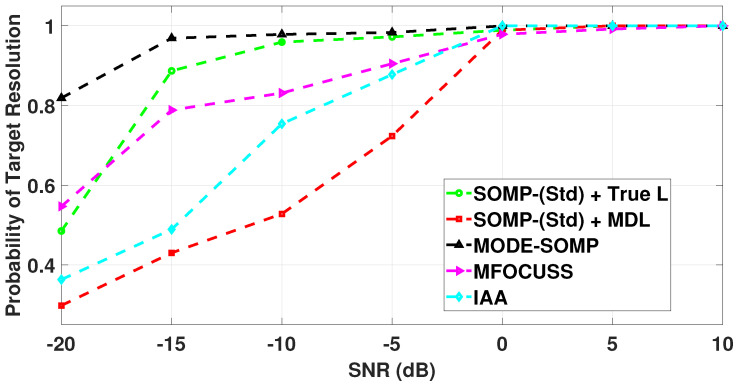
Resolution probability of DoA estimates plotted as a function of SNR for the standard and proposed algorithms. Other parameters are SNR = −20, −15, −10, −5, 0, 5, and 10 dB, and $T_{s}=80$.

The probability of target resolution is computed by dividing the number of successful resolutions with the number of Monte Carlo runs. The performance of all the algorithms is evaluated with 1000 Monte Carlo runs. The SNR values are varied from −20 dB to 10 dB and the number of snapshots is $T_{s}$ = 80 as shown in Figure 18.

From Figure 18, it can be seen that the resolution probability of all the algorithms increases with increasing SNR. The proposed MODE-SOMP algorithm is observed to have higher probability of resolving source directions at low SNR values, i.e., −20 dB to 0 dB, compared to all the other algorithms under study.

### 5.6. Time Cost of Considered DoA Estimation Algorithms

In this section we compare the computational complexity, in terms of time cost, of the proposed MODE-SOMP algorithm with that of the MFOCUSS and the IAA sparse estimator; the standard SOMP with *L* known and *L* estimated is also included for reference. Results are presented here for the scenario 2 described previously in Section 5 as a similar trend is observed for the other scenarios. This investigation was conducted using the MATLAB R2018b platform on a machine running an Intel Core i5-7200U CPU @ 2.70 GHz with 8 GB DDR3 RAM and Windows 10 Professional operating system. Using 1000 Monte Carlo runs, the average time cost for each algorithm is computed by dividing its cumulative CPU time over all runs by the number of runs [69,70]. Table 8 shows the time cost comparison of the considered algorithms. Performance is shown for both 10 dB and −15 dB SNR to represent the high SNR and low SNR regimes, respectively. As seen from the table, the average time cost of the MODE-SOMP algorithm is slightly larger than that of the standard SOMP variants but it is significantly less than the time costs of both the IAA and the MFOCUSS algorithms. Furthermore, the MODE-SOMP algorithm’s cost is similar across high and low SNRs indicating its robustness to noise. It is pertinent to mention that the second row of Table 8 shows significantly smaller time cost of the standard SOMP with estimated *L* at low SNR. However, the associated DoA estimates are not reliable due to the well-known limitation of the MDL technique of providing wrong source order estimates under low SNR conditions.

**Figure 19:**
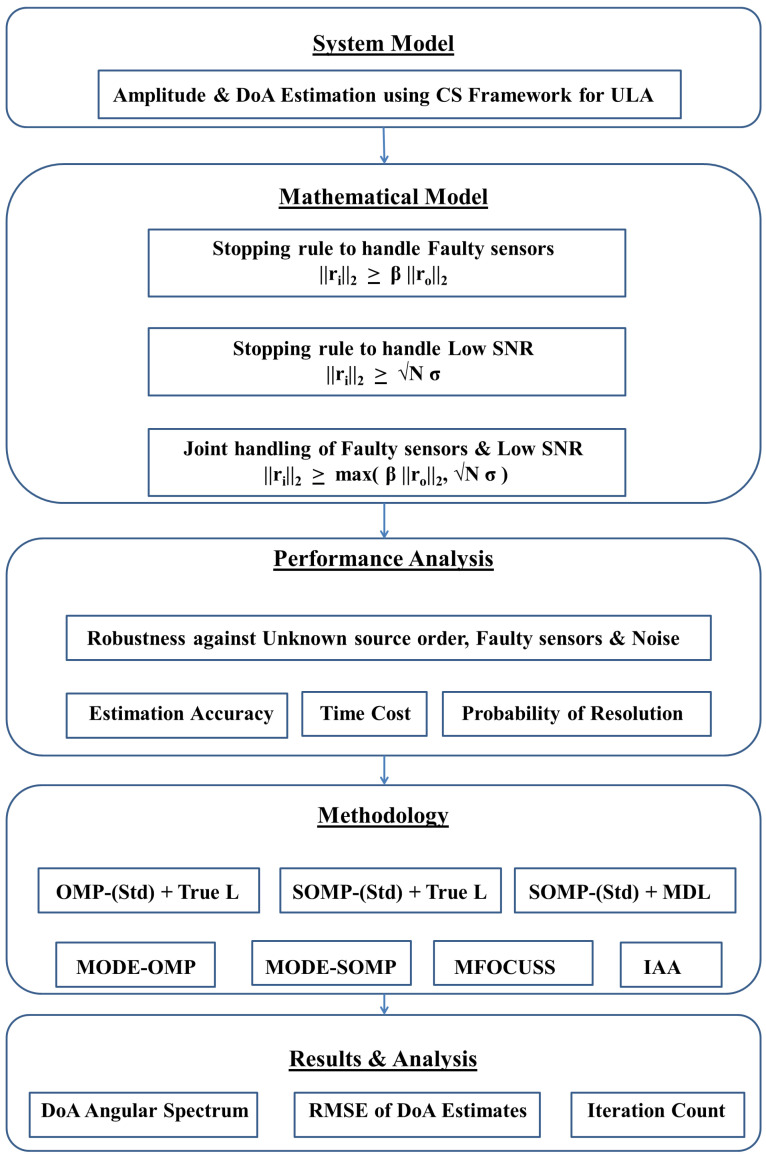
Flow chart of the study.

Figure 19 provides a flow chart of the work presented in this paper.

## 6. Conclusions

In this work modified DoA estimation algorithms within the CS framework have been proposed for a uniform linear array of underwater sensors to jointly address the practical errors including unknown source order, faulty sensors, and low SNR conditions. The presented numerical results demonstrate the superior comparative performance of the proposed MODE-OMP and MODE-SOMP algorithms in accurately estimating DoAs without prior knowledge of the source order in the low SNR regime (SNR < 0 dB), whereas at high SNRs the proposed algorithms have similar DoA estimation performance to other algorithms. Our results hold significance for practical deployments of underwater acoustic sensor arrays.

## Figures and Tables

**Table 1:** Side-lobe level increase with increasing number of faulty sensors in 24-sensor ULA.

Number of Faulty Sensors	Positions of Faulty Sensors	1st Side-Lobe Level (dB)
Nil (healthy array)	Nil (healthy array)	−13.25
1	9	−11.25
3	7, 12, 16	−9.32
6	3, 5, 9, 12, 15, 18	−7.65
10	3, 5, 7, 9, 12, 15, 16, 18, 22, 23	−5.71

**Table 2:** A sample SNR calculation for underwater ULA with 24 sensors.

Target Type	Range (Km)	SL (dB)	NL (dB)	TL (dB)	AG (dB)	SNR (dB)
Submarine	20	110	60	87	14	−23
Frigate Ship	100	135	60	102	14	−13

**Table 3:** Scenario 1: median iteration count for considered algorithms at different SNR values. Other parameter value is $T_{s}=1$.

SNR (dB)	OMP-(Std) + True *L*	MODE-OMP	IAA	MFOCUSS
10	5	5	5	48
0	5	5	5	48
−10	5	5	5	48
−15	5	5	5	48

**Table 4:** Scenario 1: median iteration count for considered algorithms at different SNR values. Other parameter value is $T_{s}=25$.

SNR (dB)	SOMP-(Std) + True *L*	SOMP-(Std) + MDL	MODE-SOMP	IAA	MFOCUSS
10	5	5	5	5	48
0	5	5	5	5	48
−10	5	2	5	5	48
−15	5	1	5	5	48

**Table 5:** Scenario 2: median iteration count for considered algorithms at different SNR values. Other parameter value is $T_{s}=25$.

SNR (dB)	SOMP-(Std) + True *L*	SOMP-(Std) + MDL	MODE-SOMP	IAA	MFOCUSS
10	5	5	4	5	48
0	5	5	4	5	48
−10	5	2	4	5	48
−15	5	1	4	5	48

**Table 6:** Scenario 3: median iteration count for considered algorithms at different SNR values. Other parameter value is $T_{s}=25$.

SNR (dB)	SOMP-(Std) + True *L*	SOMP-(Std) + MDL	MODE-SOMP	IAA	MFOCUSS
10	5	5	5	5	48
−15	5	1	5	5	48

**Table 7:** Scenario 4: median iteration count for considered algorithms at different SNR values. Other parameter value is $T_{s}=25$.

SNR (dB)	SOMP-(Std) + True *L*	SOMP-(Std) + MDL	MODE-SOMP	IAA	MFOCUSS
10	5	5	5	5	48
0	5	5	5	5	48
−10	5	2	5	5	48
−15	5	1	5	5	48

**Table 8:** Scenario 2: average time cost (in seconds) for considered algorithms at different SNR values. Other parameter value is $T_{s}=80$.

SNR (dB)	SOMP-(Std) + True *L*	SOMP-(Std) + MDL	MODE-SOMP	IAA	MFOCUSS
10	0.0074	0.0074	0.0085	0.1125	0.0694
−15	0.0078	0.0022	0.0082	0.1087	0.0836
